# Genomic-guided precision oncology in canine cancer: from comparative genomics to liquid biopsy and molecular monitoring

**DOI:** 10.4142/jvs.25370

**Published:** 2026-07-06

**Authors:** Minjeong Kang, Jaemin Kim, Jinho Park, DoHyeon Yu

**Affiliations:** 1 College of Veterinary Medicine, Gyeongsang National University, Jinju 52828, Korea.; 2 Institute of Agriculture and Life Sciences, Gyeongsang National University, Jinju 52828, Korea.; 3 College of Veterinary Medicine, Jeonbuk National University, Iksan 54596, Korea.; 4 Royal Animal Medical Center, Seoul 02140, Korea.

**Keywords:** Neoplasms, next-generation sequencing, circulating tumor DNA, minimal residual disease, precision medicine

## Abstract

**Importance:**

Traditional cancer diagnostics in veterinary medicine are limited by invasiveness and insufficient sensitivity for real-time molecular monitoring. This review critically evaluates the clinical applicability of somatic mutation profiling and liquid biopsy technologies to establish an evidence-based, tiered framework for precision oncology in canine patients, with emphasis on veterinary-specific constraints and technology selection.

**Observations:**

Key somatic biomarkers demonstrate strong clinical utility: BRAF V595E achieves > 80% diagnostic sensitivity in canine urothelial carcinoma and is detectable non-invasively in urine via droplet digital polymerase chain reaction (ddPCR). The multicenter cancer detection in dogs (CANDiD) study demonstrated circulating tumor DNA-based liquid biopsy achieves 54.7% overall sensitivity and 98.5% specificity, though stage I detection remains critically limited at 16.1%, underscoring the current gap for early-stage screening. Minimal residual disease assessment via allele-specific oligonucleotide-PCR and next-generation sequencing enables detection of molecular relapse 42–168 days before clinical manifestation in canine lymphoma. However, analytical variability across platforms, lack of standardized pipelines, and high cost represent significant barriers to routine veterinary application.

**Conclusions and Relevance:**

Genomic-guided oncology offers substantial clinical value for canine cancer management yet requires veterinary-specific validation before broad adoption. Targeted sequencing panels currently represent the optimal balance of cost-effectiveness and clinical utility for most veterinary practitioners, while ddPCR is preferred for longitudinal tracking of known mutations. Liquid biopsy cannot serve as a standalone screening tool at present, but functions as a valuable complementary monitoring approach. Continued canine-specific biomarker research and multicenter data sharing are essential prerequisites for integrating precision medicine into routine veterinary care.

## INTRODUCTION

Cancer represents a major health challenge and is a leading cause of death in humans and dogs. Although incidence and mortality rates vary among species, cancer-related deaths account for 15%–25% even in species less affected by neoplastic diseases [1], highlighting the need for advanced technologies in canine cancer care.

Dogs develop cancer spontaneously and share biological parallels with humans regarding tumor biology, including shared oncogenic pathways, mutational landscapes, and therapeutic responses. These similarities position canine cancers as valuable comparative models that can provide bidirectional translational insights, advancing veterinary cancer care and contributing to human oncology research [2].

Recent advancements in genomic technologies have fundamentally transformed our understanding of cancer as a genetic disease, allowing for deeper insights into its molecular underpinnings. Cancer diagnosis relies on histopathology and imaging modalities, which are often limited by high invasiveness and low sensitivity. Moreover, standard cancer therapies are frequently associated with drug resistance and severe adverse effects. These limitations prompted the emergence of personalized medicine, which focuses on the role of genomics in driving the development, progression, and treatment responsiveness of tumors [3]. Somatic mutation analysis and liquid biopsy are promising molecular-based approaches for cancer care in veterinary oncology, although their clinical application remains limited.

Despite advances in diagnostic imaging and histopathology, veterinary oncology faces persistent challenges in real-time disease monitoring, early relapse detection, and treatment response assessment. Conventional methods, such as measuring lymph node size or imaging-based tumor evaluation, often detect disease progression only after substantial tumor burden accumulation. This reactive approach limits opportunities for timely therapeutic intervention and fails to capture the dynamic molecular evolution of tumors during treatment.

Liquid biopsy and minimal residual disease (MRD) assessment represent transformative approaches that address these limitations. By detecting tumor-derived molecules—particularly circulating tumor DNA (ctDNA)—in body fluids such as blood, urine, or effusions, these noninvasive techniques enable serial molecular monitoring with unprecedented sensitivity. MRD assessment, which detects malignant cells below conventional detection thresholds, has become a cornerstone of precision hematologic oncology in humans, guiding treatment intensity decisions and predicting relapse months before clinical manifestation.

The landscape of veterinary oncology has been significantly shaped by recent comprehensive reviews. Chibuk et al. [4] established the foundational potential of liquid biopsy across various clinical use cases, while Domrazek and Jurka [5] provided a detailed technical compendium of next-generation sequencing (NGS) methodologies available to researchers. Furthermore, Arendt et al. [6] synthesized the complex interplay between germline predispositions and somatic landscapes. However, a critical synthesis that translates these high-level genomic data into a tiered, actionable framework for the practicing veterinarian remains absent.

Unlike previous works that offer a pan-cancer descriptive overview, this review provides a critical appraisal of platform-specific analytical variability and the biological constraints unique to canine patients—such as breed-specific genetic architecture and species-distinct ctDNA shedding kinetics. By introducing a tiered framework for technology selection (targeted Sequencing vs. digital droplet polymerase chain reaction [ddPCR] vs. whole-genome sequencing [WGS]), this review aims to guide evidence-based clinical decisions in various practice settings, from resource-limited clinics to advanced referral centers.

## METHODS

This review establishes the foundational principles of canine cancer genomics essential for precision oncology. We examine the biological basis of cancer as a genomic disease, explore current technologies for mutation detection, and critically analyze the comparative landscape of canine and human cancer genomics. Key somatic mutations with diagnostic, prognostic, and therapeutic implications in major canine tumor types will be highlighted.

Moreover, the application of somatic mutation profiling and liquid biopsy technologies in veterinary oncology will be explored, with particular emphasis on hematologic malignancies including lymphoma. We detail how high-sensitivity detection platforms enable MRD monitoring, treatment response assessment, and early relapse prediction. We examine current evidence from canine studies, including the multicenter CANDiD Study and tumor-specific liquid biopsy applications. Finally, we discuss the integration of artificial intelligence (AI) and data-sharing initiatives that will accelerate biomarker discovery and clinical validation.

### Declaration of AI and AI-assisted technologies in the writing process

The authors declare that no generative AI was used to generate the content of this manuscript. However, Google Gemini was utilized to produce Fig. 1.

**Fig. 1:**
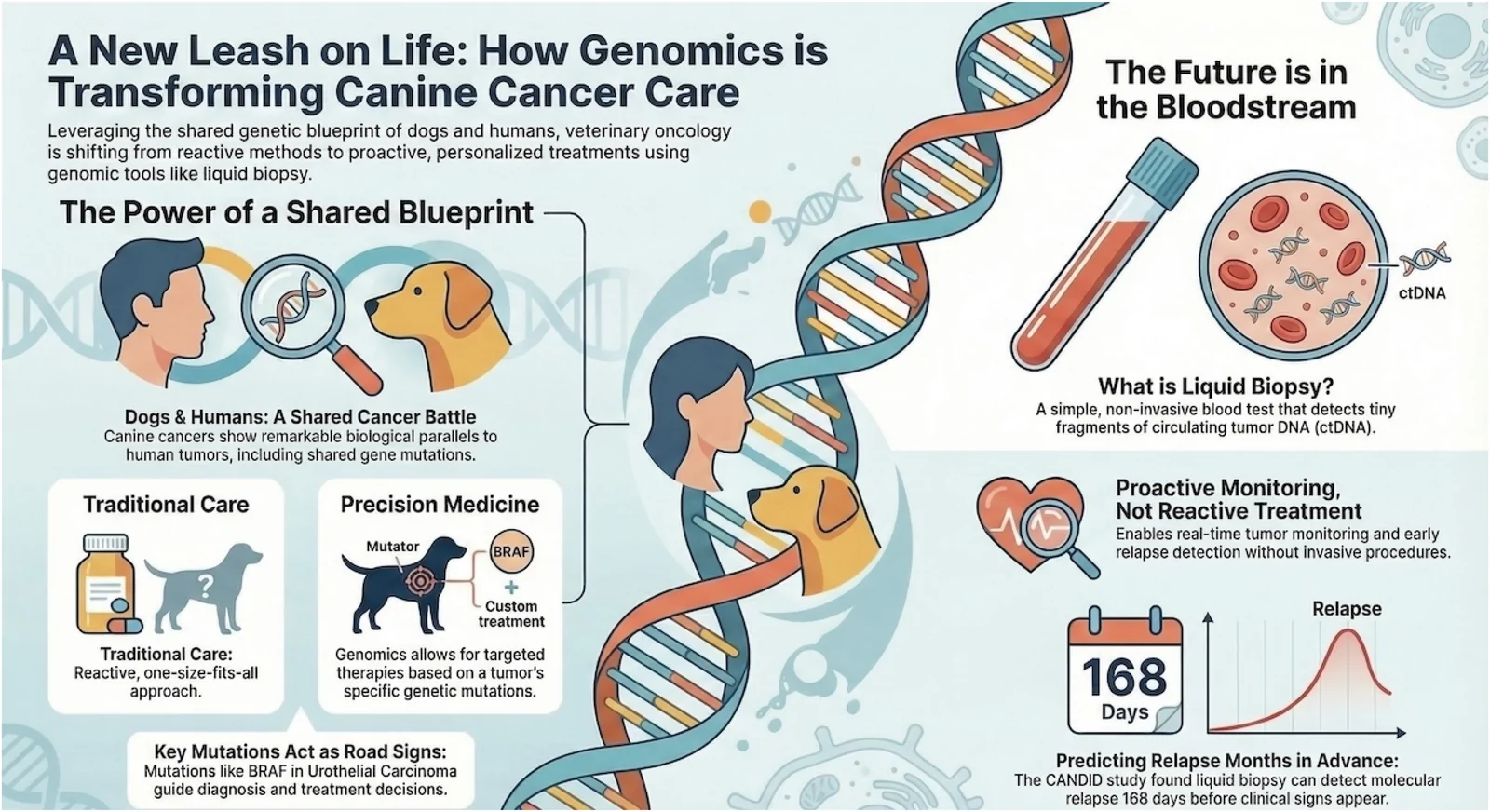
Paradigm shift toward precision medicine via comparative genomics. Human and canine cancers share biological parallels, including oncogenic pathways and mutational landscapes. Traditional reactive care is transitioning to genomics-guided precision medicine. Non-invasive liquid biopsy of circulating tumor DNA facilitates proactive monitoring, with molecular relapse detectable up to 168 days before clinical signs.

## OBSERVATIONS

### Cancer as a genomic disease

Cancers are caused by genetic mutations in oncogenes and tumor suppressor genes. These mutations are inherited or accumulate over time, and as few as three sequential mutations can initiate tumorigenesis [7]. Mutations are continuously generated by exogenous and endogenous factors, including DNA damage, replication errors, and defects in DNA repair. Mutations include base substitutions, small insertion–deletions (indels), and structural variants (SVs), including genome rearrangements and changes in chromosome copy number. Epigenetic alterations like DNA methylation and histone modification also play a crucial role in tumor development [8]. In a study of mutations in 23,829 tumor samples, the most common mutation type was single-nucleotide variants, whereas indels (mutations of < 50 base pairs) accounted for ~10% [8].

#### Oncogenes and tumor suppressor genes

Among the diverse mechanisms driving tumorigenesis, genomic alterations that induce sustained proliferative signaling and evade growth suppression have been central to the discovery of genomic cancer biomarkers [9]. Oncogenes are genes that promote abnormal, sustained cell proliferation through gain-of-function mutations or overexpression of proto-oncogenes, which normally regulate cell growth and differentiation. Due to mutations in receptors or downstream signaling pathways, oncogenes can remain active even in the absence of ligands. In contrast, tumor suppressor genes inhibit cell division or eliminate damaged cells, thereby preventing tumor formation. In their case, loss-of-function mutations can cause cancer development. For example, *TP53* induces cell cycle arrest or apoptosis, and inactivation of this pathway allows abnormal cells to evade elimination and continue proliferating [9].

#### Somatic mutations in and beyond coding regions

Historically, most oncogenes and tumor suppressor genes have originated from protein-coding regions, as these regions were the focus of oncology research due to their direct transcription into mRNA that directs protein synthesis. However, noncoding regions play critical roles in transcriptional regulation, RNA processing and stability, chromatin interactions, and splicing, thereby controlling gene expression levels. Noncoding regions—such as promoters, enhancers, introns, untranslated regions, and topologically associated domains—comprise > 98% of the genome. Mutations in these regions can influence gene expression by altering the number of specific proteins and can contribute to the production of aberrant proteins, necessitating further research [10] and emphasizing the need for WGS [11].

Ongoing efforts to uncover such key mutations have led to the discovery of variants that are now utilized in clinical practice as important biomarkers for diagnosis, monitoring, selecting targeted therapies, and predicting drug resistance.

#### Methods to detect somatic mutations

Diagnostic methods for detecting mutations can be broadly categorized into NGS- and PCR-based approaches. NGS-based methods include targeted sequencing (TS), which analyzes specific genomic regions with well-established pathogenic mechanisms and clinical significance; WGS, which comprehensively analyzes the entire genome; and WES, which focuses solely on exonic regions [12,13]. As nontargeted approaches, WGS and WES offer the advantage of detecting unknown or rare mutations and are capable of simultaneously identifying various types of mutations beyond single-nucleotide variants. WGS particularly excels at detecting driver mutations in noncoding regions and SVs, thereby enabling a more comprehensive understanding of the cancer genome. However, WGS has several limitations, including high cost, long turnaround time, the need for substantial computational resources and storage, challenges in interpreting the functional impact of noncoding variants and structural rearrangements, and a lack of standardized analysis algorithms. Moreover, WGS has a lower sensitivity than that of TS, making it unsuitable for repeated testing and detection of specific target mutations, during recurrence or metastasis monitoring, especially after resection of the primary tumor [13].

TS can be divided into amplicon-based and hybrid capture-based methods; the former is more cost-effective and has a higher on-target read rate, whereas the latter provides more uniform coverage [13]. Compared with non-TS, TS generates smaller data volumes and allows for simpler analysis, with advantages including higher sensitivity, shorter turnaround time, and lower cost. TS also enables a deeper sequencing coverage than that of WGS. However, TS is limited to detecting only known mutations, and its coverage depends on the gene panel design. Nevertheless, the ability to analyze multiple gene mutations simultaneously using such panels provides a practical benefit [12].

SVs, genomic alterations of > 50 base pairs, are often difficult to detect using conventional short-read sequencing technologies. To overcome this limitation, long-read sequencing (LRS) has emerged as an advanced NGS technique. LRS enables the more accurate detection and characterization of SVs, including indels, inversions, duplications, and translocations. Two primary analytic approaches are used for SV detection. Most studies rely on mapping the reads to a reference genome to identify SVs based on split-read alignments, due to its greater practicality; other studies perform de novo assembly of long reads, followed by comparison to the reference genome. LRS has been applied to various cancer types, often in combination with short-read sequencing, and for detecting SVs in liquid biopsy specimens [14].

High-throughput chromosome conformation capture sequencing (Hi-C) enables analyses of the three-dimensional structure of the genome, providing a favorable approach for detecting tumor purity, copy number variants, and chromosomal rearrangement in cancer tissues. Compared with WGS, which requires high sequencing coverage and struggles to detect breakpoints in repetitive regions where chromosomal rearrangement frequently occurs, Hi-C is more effective for identifying large-scale chromosomal rearrangements [15].

PCR-based techniques include allele-specific oligonucleotide (ASO)-PCR, real-time quantitative PCR (qPCR), BEAMing, and ddPCR [16]. Among these, ddPCR is a third-generation PCR method that amplifies DNA by partitioning it into over 20,000 lipid-based microdroplets. Unlike conventional qPCR, which is less effective for detecting rare mutations and prone to inconsistent results due to varying sample contamination levels, ddPCR offers superior sensitivity, reproducibility, and resistance to PCR inhibitors [17,18]. Furthermore, ddPCR allows absolute quantification, enabling the determination of the precise copy number of the target DNA. These characteristics enable the quantification of tumor mutations, even in highly diluted body fluids like blood and urine. Compared with NGS-based TS, ddPCR is more sensitive in detecting diluted genetic variants in small-volume clinical samples, and it can calculate absolute copy numbers and mutation frequencies with single-molecule sensitivity. However, unlike NGS, ddPCR is limited in its multiplexing capacity and does not provide information about functional sequelae of the detected mutations [19]. Therefore, PCR-based methods, including ddPCR, are well-suited for tracking selected mutation markers following mutation screening by NGS and are highly effective for the repeated quantification of specific targets. Currently, no clear guidelines exist on which platform to select, and researchers and clinicians must find an appropriate balance by considering factors like cost, accuracy, analytical capability, and technical complexity. TS requires the establishment of clinical reliability and reproducibility, as the lack of standardized analytical pipelines can result in inconsistent outcomes across laboratories, even when using the same dataset [13]. Thus, no diagnostic method can be deemed universally superior, and the appropriate technique should be selected based on the clinical objective and context.

Critical evaluation of platforms for veterinary application: While TS panels are cost-effective and offer practical multi-gene analysis, their reliance on pre-defined panel designs—primarily developed from human tumor data—may miss rare or canine-specific driver mutations. Across laboratories, the lack of standardized analytical pipelines for TS can result in discordant variant calls even from identical datasets, a critical limitation for clinical decision-making [13]. Validation studies specifically in canine tumor types remain sparse, and clinical performance metrics (sensitivity, specificity, positive predictive value) have not been systematically established for most commercial veterinary panels.

Conversely, ddPCR provides superior absolute quantification and single-molecule sensitivity for known targets, making it particularly well-suited for ctDNA monitoring in liquid biopsy applications. However, ddPCR fails to capture clonal evolution—a critical limitation during long-term monitoring of hematologic malignancies where subclonal emergence is frequent. Its low sample throughput and complex workflow also limit scalability in high-volume clinical settings.

WGS, while comprehensive, has a lower sensitivity than TS for detecting specific target mutations at low allele frequencies, making it unsuitable for post-treatment MRD monitoring. The high cost (typically USD 1,000–3,000 per sample), long turnaround time (2–4 weeks), and absence of veterinary-validated bioinformatics pipelines further restrict its routine clinical use.

#### Current limitations of mutation analyses

Although the association between mutations and tumors is increasingly well-established, detecting driver mutations remains challenging. For example, *TP53* is frequently mutated, but under the 20/20 rule, a gene must have ≥ 20% recurrent missense mutations or ≥ 20% inactivating mutations to be classified as a driver [20]. Network analysis [20], RNA sequencing combined with functional expression data [21], and matched germline analysis to exclude incidental germline variants [22] are critical to distinguish drivers from passengers.

However, when mutations are located in noncoding regions, these validation processes become more difficult. Reports on noncoding mutations have only recently become more frequent, and current analyses rely on the criteria originally developed for coding mutations. These criteria mainly require two evidence types: a positive selection signal indicating that the mutation occurs more frequently than expected by chance and a predicted functional impact of the mutation. As noncoding regions remains poorly studied, distinguishing meaningful and passenger mutations is more difficult for noncoding than for protein-coding regions [10,23]. Attempts have been made to apply the ACMG/AMP guidelines, but interpretation standards are either not yet fully established or are being continuously revised, which has led to a generally conservative approach [11]. Moreover, tumors are inherently heterogeneous, and mutations are not uniformly distributed across the genome and tend to occur frequently in certain regions. This implies that a biopsy may not sufficiently represent the entire genetic profile of a tumor [23]. Additionally, mutation rates can vary depending on transcription and replication activity. For example, highly transcribed genes are more likely to undergo DNA repair, and genes that replicate early tend to accumulate fewer mutations. Therefore, using mutation frequency as the only criterion may lead to misinterpretation. Furthermore, functionally high-risk mutations that decisively influence tumor development often occur at low frequencies, making them inherently difficult to detect [11]. These limitations in mutation detection apply to humans and dogs, and in the relatively under-researched field of veterinary medicine, these key challenges must be addressed.

#### Platforms enabling longitudinal clinicogenomic analyses

With accumulating sequencing data, the availability and accessibility of cancer genomic analyses have expanded. In parallel, platforms capable of integrating these data with clinicopathologic information are gaining prominence. cBioPortal (https://www.cbioportal.org) visualizes The Cancer Genome Atlas (TCGA) datasets and enables longitudinal analysis by supporting the integrated evaluation of genomic and clinical data. In lung cancer, the analysis of genomic data before and after crizotinib treatment has revealed an increase in *ALK* mutations, confirming the presence of resistance mutations. Such open-source, web-based platforms are expected to serve as powerful tools in precision oncology, facilitating the development of personalized therapies [24].

### Comparative oncology: human and canine cancer genomics

Canine tumors are naturally occurring animal models used in human oncology research. Dogs develop the same types of cancers as humans, and because of similar histopathological features, many classifications of canine tumors have been adapted from those of human cancers. Humans and dogs exhibit similarities in gene and somatic driver mutations and in tumor biology, including gene targets, drug responses, and resistance mechanisms [2]. Studies using dogs as models have provided critical insights into canine cancers. Mutation analyses of various canine tumors are being expanded through comparative studies with human cancers. In representative large-scale studies using TS, mutations in tumor suppressor genes and oncogenes, which are well-characterized in human cancers, were frequently observed in canine tumors [25,26]. One study analyzed genomic data from 1,108 dogs and evaluated 48 coding mutations commonly reported in human cancers, identifying several mutations frequently present in canine tumors and associated with clinical prognosis [25]. Among the tumor types included in their study, hemangiosarcoma (the most common type; 19% of all samples) was enriched with *PIK3CA* and *NRAS* mutations. Across all tumors, *PIK3CA* and *TP53* mutations were associated with poor prognosis, whereas *NRAS, KIT*, and *ATM* mutations were associated with favorable outcomes. Another study analyzed 59 coding mutations commonly observed in human cancers across 671 dogs and 23 tumor types. *TP53* showed the highest mutation frequency at 22.5%, followed by *ARID1A* and *PIK3CA* [26]. In pulmonary adenocarcinomas and urothelial carcinomas (UCs), *ERBB2* and *BRAF* mutations were detected at high frequencies of ~50% and ~40%, respectively. Consistent with previous reports, *TP53* and *PIK3CA* mutations were significantly more enriched in sarcomas than in carcinomas. These two studies demonstrated that tumor suppressor genes and oncogenes considered clinically important in humans are also present at high frequencies in canine tumors, indicating the similarities in genetic and functional pathways in human and canine cancers. Thus, canine cancer research has clinical utility in tumor diagnosis and classification, potential therapeutic target identification, and drug response prediction.

However, these studies focused solely on well-established mutations in human oncology. Consequently, unique mutational patterns that may play key roles in specific canine tumors remain unknown. For example, although *PIK3CA* mutations are frequent in humans and dogs and occur at a high frequency in canine hemangiosarcoma, the incidence and anatomical predilection of this tumor differ substantially between the two species. In humans, angiosarcoma is extremely rare, accounting for ~0.01% of all cancers [27], whereas it accounts for ~2% of all tumors and represents 45%–51% of splenic tumors in dogs [28]. Despite overall genetic similarities, mutation frequencies and tumorigenic mechanisms differ between tumor subtypes. In humans, *BRAF* mutations are a key oncogenic pathway, with MAPK signaling activation by *BRAF* mutations considered central to tumor progression. The association between ultraviolet (UV) exposure and *BRAF* mutations is well-established, and non-UV-associated melanoma is rare [29]. However, *BRAF* mutations in canine melanoma have a low frequency, observed in ~6% of melanomas and 17% of melanocytomas [30], and the relationship with UV exposure remains elusive [29]. Interestingly, the *BRAF* V595E mutation is highly prevalent in canine UC (67%) and prostatic carcinoma (80%) and is currently used as a diagnostic biomarker for UC [30]. However, *BRAF* mutations are rare in human bladder cancer and are not widely used as diagnostic or therapeutic targets [29].

This cross-species similarity extends beyond solid tumors and has been observed in hematologic malignancies. Lymphoma, which accounts for more than 80% of hematologic malignancies in dogs, has been studied in the field of comparative oncology based on its numerous molecular similarities. Diffuse large B-cell lymphoma (DLBCL), the most common hematologic cancer in humans and dogs, shares similar molecular signaling pathways, leading to many studies that have utilized dogs as preclinical models for human DLBCL [31]. Human DLBCL is subclassified into activated B-cell type (ABL), germinal center B-cell-like (GCB), and primary mediastinal DLBCL, based on molecular features, with each subtype exhibiting distinct genetic alterations, oncogenic mechanisms, prognoses, and therapeutic responses; however, such a molecular subtyping system has not yet been established in dogs [32]. Signaling pathways commonly identified in canine and human DLBCL include nuclear factor (NF)-κB, PI3K–AKT, JAK–Stat, p53, interleukin (IL)-6, IL-10, and IL-2 signaling, all of which are involved in the immune regulation, proliferation, and survival of tumor cells. In particular, dysregulation of the NF-κB pathway is a major mechanism contributing to tumorigenesis and treatment resistance in human ABL, and the predominant activation of the canonical NF-κB pathway observed in canine DLBCL supports this biological similarity [33,34,35]. The frequent identification of loss-of-function mutations in the tumor suppressor gene *TRAF3*, a negative regulator of NF-κB, in canine B-cell lymphoma further supports this similarity [36,37]. Genetic similarities of canine T-cell lymphoma and human peripheral T-cell lymphoma (PTCL) have also been reported. Mutations in upstream signaling regulators of the MAPK pathway, including *MET*, *KDR (VEGFR)*, *STK11*, and *BRAF*, commonly observed in human tumors, have been identified in canine T-cell lymphoma, suggesting the potential of the MET–ERK axis as a novel anti-cancer target model [38].

However, certain aspects of hematologic malignancies in dogs do not align with those in humans. For example, T-zone lymphoma, the most commonly reported subtype of indolent nodal lymphoma in dogs, is extremely rare in humans and is considered a morphological variant of PTCL not otherwise specified, rather than an independent subtype [39]. Follicular lymphoma, an indolent B-cell lymphoma that is the second most common lymphoma in humans after DLBCL, has a frequency of <1% among hematologic malignancies in dogs, making it extremely rare [40]. Although dogs and humans share molecular signaling pathways, differences exist in specific mutations and copy number alterations [41]. For instance, *TRAF3* mutations were detected in 44% of 84 canine DLBCL samples, making it the most frequently observed alteration, whereas its frequency was ~9% in 148 human DLBCL samples [36]. *EZH2*, frequently mutated in the human GCB subtype and a key oncogenic driver as well as a therapeutic target, is rarely detected in dogs [36,42]. The *H3K27M* mutation—associated with poor prognosis in canine DLBCL—is a characteristic mutation in human brain tumors but rare in human DLBCL [37]. Regarding T-cell lymphoma, recurrent mutations in genes like *PTEN*, *SATB1*, and *SETD2*, which have conserved functions in T-cell tumorigenesis, have been identified in both species. Other gene mutations, including those in the NLRP family, *TBC1D26*, and *COX8A*, have only been reported in dogs, whereas canonical histone- and methyltransferase-related mutations commonly observed in human lymphomas are rarely found in dogs [35].

Differences between dog breeds are also noteworthy. These differences can be pronounced in purebred dogs in closed breeding systems, leading to breed-specific tumor susceptibilities and genetic risk factors. Examples include the high incidence of gastric cancer in Belgian breeds, histiocytic sarcoma in Bernese Mountain Dogs, and invasive transitional cell carcinoma in Scottish Terriers and Shetland sheepdogs [43]. The *NIPAL1* G256D germline mutation, along with other cancer-related somatic mutations, predicts the genetic risk of invasive urinary carcinoma in Shetland Sheepdogs, with a point mutation in the *PIPAL1* gene increasing the risk by eightfold [44]. Genetic differences across breeds have also been reported in hematologic malignancies. Although B-cell lymphomas show a high proportion of shared mutated genes across breeds, T-cell lymphomas exhibit only a 15% overlap in mutation profiles between Boxers and Golden Retrievers. Lymphoblastic PTCL, which shows aggressive clinical progression, is commonly observed in Boxers and is closely associated with mutations in the PTEN–mTOR signaling pathway. In contrast, Golden Retrievers are predisposed to T-zone lymphoma, which is characterized by a relatively low mutation burden and a favorable prognosis [35].

Although canine and human cancers share many genetic mutations and oncogenic pathways, considerable differences exist in tumor subtype characteristics across species and breeds. Considering that the frequency and function of key gene mutations identified in specific tumor types may differ between humans and dogs, identifying mutation biomarkers specific to canine cancers and clinically validating them should be emphasized. Such canine-specific biomarker research is valuable for diagnostic purposes and serves as an essential foundation for therapeutic target selection, treatment response monitoring, and disease recurrence surveillance (Fig. 1).

### Somatic mutations in canine solid tumors: diagnostic, prognostic, and therapeutic biomarkers

This section aims to explore the key somatic mutations currently applied in clinical settings for canine solid and hematologic tumors, along with their clinical implications.

#### Multigene panel and targeted therapy

As part of the current increase in precision medicine, NGS-based tumor mutation profiling using multigene panels, including SearchLight DNA (SearchLight DNA™; Vidium Animal Health, USA), FidoCure (FidoCure®; The One Health Company, USA), and OncoK9 (OncoK9®; PetDx, USA), is increasingly implemented. FidoCure utilizes a panel of 56 commonly known coding region tumor genes, aiming to identify actionable mutations and guide targeted therapy. SearchLight DNA analyzes 120 tumor-associated genes and classifies biomarkers according to their diagnostic, prognostic, or therapeutic relevance [45]. PetDx has uniquely contributed to the field by publishing studies on multicancer early detection tests, primarily based on liquid biopsy. These efforts collectively contribute to the expansion of precision oncology through comprehensive pan-cancer genomic profiling.

Growing evidence in human oncology has demonstrated that the selection of targeted therapies is increasingly based on the presence of specific mutations rather than the anatomical tumor location. However, only a few studies have described the use of targeted therapies based on somatic mutations. Studies using the FidoCure platform reported improved prognoses in dogs treated with lapatinib for *BRAF*-mutant tumors, trametinib for *ARID1A* mutations, dasatinib for *BRCA1* mutations, and rapamycin for *PIK3CA* and *TP53* mutations in dogs with splenic hemangiosarcoma [25,46]. Targeted therapy guided by genetic mutations identified through SearchLight DNA has been associated with improved progression-free survival (PFS) [47]. Although study numbers remain limited, the importance of research on tissue-agnostic therapy is increasingly recognized in veterinary oncology.

Following multigene panel studies, increasing attention has been directed toward identifying mutation biomarkers for the diagnosis, prognosis, and therapy of individual tumor types; however, clinically applicable markers remain rare. The following sections analyze three representative tumor types: canine mast cell tumor (MCT), UC, and canine mammary tumor (CMT).

#### MCT: prognostic implications of c-kit mutations and tyrosine kinase inhibitor (TKI) resistance

MCTs account for 7%–12% of cutaneous tumors in dogs. *c-kit* mutations in exon 11 serve as prognostic indicators as they are associated with a poor prognosis, high histologic grade, and aberrant KIT protein localization [48]. Dogs with *c-kit* mutations in exon 8 showed a 70% lower risk of death than that in those without; thus, this mutation is associated with a favorable prognosis. These dogs typically exhibited low-grade MCT, a lower frequency of aberrant KIT expression patterns, and lower Ki67 indexes and AgNOR scores [49]. Abnormal cytoplasmic expression of KIT protein, particularly pattern 3 compared with pattern 1, has been linked to reduced TKI responses. One possible explanation is that secondary mutations arising after TKI administration may alter the amino acid sequence of the drug-binding site or reduce binding affinity [50]. However, some studies have reported that dogs with *c-kit* mutations had shorter PFS and lower response rates than that in those with wild-type *c-kit*. In a study evaluating toceranib treatment for canine MCT, no significant prognostic difference was observed based on mutation status [51], suggesting that the presence of *c-kit* mutations may not always predict TKI responsiveness. Although *c-kit* mutation status may provide useful prognostic information regarding drug response, prospective studies involving larger cohorts are necessary to validate its clinical utility.

#### UC: *BRAF* V595E mutation and liquid biopsy

This *BRAF* mutation in UC is a widely used example of mutation detection for diagnostic purposes. This mutation is detected in > 80% of canine UC cases and demonstrates a high diagnostic rate for this tumor type [30]. A major clinical advantage of *BRAF*-TS is its applicability to liquid biopsy, as it can be detected in urine samples containing tumor-derived DNA, allowing for a noninvasive, convenient diagnostic method. In a study using ddPCR, the mutation was detected in 75% of dogs with UC and in 85% of those with prostatic carcinoma [52]. The CADET BRAF test (CADET® BRAF assay; Sentinel Biomedical, USA), available in clinical settings, analyzes *BRAF* mutations or copy number variants from free-catch urine samples, offering 95% diagnostic accuracy with a very low detection threshold, capable of identifying as few as 10 mutation-bearing cells. This test can be used for diagnosing, evaluating treatment responses, monitoring recurrence, identifying high-risk individuals, and enabling early detection.

Subsequent studies have attempted to detect *BRAF* mutations in plasma. In one study, ctDNA in plasma was detected in nine of 11 dogs with tissue-confirmed *BRAF* mutations; in the remaining two dogs, ctDNA was later detected as the disease progressed. The concentration of *BRAF*-mutated ctDNA was significantly higher in dogs with this mutation than in wild-type dogs (14.05 ± 13.51 ng/mL vs. 0.21 ± 0.41 ng/mL). Dogs with ctDNA concentrations above 8.6 ng/mL had significantly higher mortality, indicating the potential utility of plasma-based ctDNA measurements for UC diagnosis and prognosis [52]. Moreover, ctDNA levels changed in response to surgical resection or treatment, supporting the value of ctDNA as a tool for tumor monitoring. Therefore, the *BRAF* mutation in canine UC represents an example of a single-gene biomarker that can be used for diagnostic and prognostic evaluations. However, in the aforementioned study, plasma ctDNA levels did not significantly differ according to tumor stage or metastatic status, suggesting that ctDNA levels may not fully reflect tumor burden dynamics. Furthermore, low-level ctDNA was detected in one wild-type dog, which may indicate false positives in liquid biopsies [53]. *BRAF* mutation has also prompted the exploration of *BRAF*-targeted therapies in dogs with UC. A study on vemurafenib, an agent known for its high initial response rate in human metastatic melanoma, demonstrated favorable outcomes (a 36% response rate and median PFS of 6 months) in dogs with UC harboring *BRAF* mutations compared with those after other single-agent therapies. Similar to humans, dogs showed mechanisms of resistance, such as MAPK pathway reactivation and activation of alternative pathways including PI3K–AKT, JNK, and mTOR [54].

#### CMT: relevance of *PIK3CA, BRCA2*, and poly ADP ribose polymerase (PARP) inhibitors

CMTs, a common tumor in intact female dogs, are characterized by aggressive progression and poor prognosis in histologically malignant subtypes. These tumors often exhibit high metastatic potential and low chemotherapy responsiveness, complicating their management beyond surgical excision and highlighting the need for new therapeutic strategies [55,56].

Due to biological and molecular pathological similarities, spontaneously occurring CMTs are considered relevant models for human breast cancer, and sequencing studies of tumor tissues are ongoing. In one WES study of 20 matched CMT and healthy tissues, *PIK3CA* mutations were identified in 45% of cases. Notably, the *PIK3CA* H1047R (A3140G) mutation, a known hotspot in human breast cancer, was found in 18 of 62 CMT samples, indicating the conservation of the same hotspot mutation between species [57]. Additionally, *BRCA1* and *BRCA2* mutations are associated with CMT development and demonstrate potential as biomarkers and therapeutic targets [58]. *BRCA2* is an essential gene that facilitates the accurate repair of double-strand DNA breaks by promoting RAD51 recruitment and filament formation. Loss of BRCA1 or BRCA2 function impairs RAD51 complex assembly, increasing genomic instability [59], chromosomal aberrations, and tumorigenesis. In humans, women with *BRCA1* or *BRCA2* mutations are at significantly increased lifetime risk of developing breast and ovarian cancers; thus, these mutations are targets for cancer screening. In CMT, mutations have been identified within the BRC repeat region in exon 11 of the *BRCA2* gene, which mediates RAD51 binding, as well as in the C-terminal domain [58,60]. In dogs, the expression levels of RAD51 and BRCA1 are correlated with tumor malignancy and lymph node metastasis [60]. In humans, PARP inhibitors such as olaparib and talazoparib have been approved for the treatment of metastatic breast cancer in patients with *BRCA* mutations, and these therapies improve survival and quality of life compared with those after conventional monotherapy. Even among patients without germline mutations, those with somatic *BRCA1/2* mutations have a 50% response rate and a 67% clinical benefit rate [61]. No study has confirmed the efficacy of PARP inhibitors in CMTs with *BRCA* mutations; however, considering the genetic similarity between species, their clinical applicability should be evaluated. In humans, some patients with *BRCA* germline mutations do not respond to PARP inhibitors, and as the average response duration is only ~6 months, resistance mechanisms are currently being investigated. The current lack of biomarkers that can accurately identify patients with a high likelihood of response to PARP inhibitors suggests that veterinary oncology may face the same challenges [61].

Despite these promising similarities, direct translations between CMT and human breast cancer are limited by molecular, hormonal, and breed-specific differences. Molecular and histologic profiles are similar but not identical in both species [62,63]. Hormonal exposure and receptor patterns also differ: female dogs experience lower lifetime estrogen exposure than that in women [64], and high ERα/PR expression is largely confined to benign or low-grade CMTs, whereas human breast cancers commonly express ERα/PR across malignant grades, defining key molecular subtypes and therapeutic targets [65,66]. Breed-dependent risk in dogs adds another layer of variability [67]. Therefore, adopting human breast cancer biomarkers and targeted therapies like PARP inhibitors via comparative oncology is highly valuable, yet demands further research.

### Somatic mutations in canine hematologic tumors: diagnostic, prognostic, and therapeutic biomarkers

#### Lymphoma-associated mutations and clinical implications

Currently, monitoring of chemotherapy success relies on measuring superficial lymph node size with imaging modalities [68], while data on relapse surveillance after remission, prognosis based on genetic mutations, and chemotherapy selection are extremely limited. Furthermore, there is a lack of established criteria to identify individual variations in drug resistance or prognosis, not only among patients with the same cancer type but also within the same individual, where therapeutic responses can evolve over time. DLBCL, the most common subtype in dogs, shows the highest response rate and longest remission duration with multi-agent therapy (the CHOP protocol), making it the most widely used regimen, but most patients eventually experience rapid relapse and develop chemotherapy resistance [69]. Therefore, in addition to molecular subtyping based on genetic alterations, molecular monitoring tools like MRD assessment are needed to sensitively assess treatment response and relapse risk.

Although cytological examination alone can currently distinguish lymphoma from reactive hyperplasia with a sensitivity and specificity of > 90% [70], studies on specific genetic biomarkers that enable subtype classification and prognostic prediction at the molecular level are scarce. Based on specific gene mutations recurrently observed in canine lymphoma, diagnostic and prognostic markers or therapeutic targets specific to lymphoid neoplasms beyond pan-cancer gene profiling have been established. For example, in the K9 Lymphoma Assay, a TS panel study, *TRAF3*, *POT1*, *FBXW7*, *MYC*, and *DDX3X* mutations were detected, consistent with previous reports. *TP53* and *SETD2* mutations were associated with poor survival, whereas *TBLXR1* mutations were associated with better survival, showing their prognostic value in B-cell lymphomas. In contrast, these mutations were not associated with prognosis in T-cell lymphomas [71]. Another study identified recurrent *TP53* mutations across various anatomical sites, and the presence of mutations was associated with chemotherapy response and survival duration [72]. These studies suggest that molecular-level mutation analysis may enable more refined subtype classification and patient-specific prognostic prediction in canine lymphoma. Future clinical goals in veterinary oncology should include defining therapeutic targets, monitoring treatment responsiveness, determining relapse risk early using mutation-based biomarkers, and establishing a liquid biopsy-based longitudinal monitoring system.

#### MRD assessment in hematologic malignancies

Although mutation-based biomarkers are critically involved in diagnosis, prognostication, and therapeutic decision-making, their application for monitoring disease dynamics, particularly detecting low-level residual disease, requires more sensitive and standardized biomarkers and detection techniques. In hematologic malignancies, which progress rapidly and carry a high recurrence risk, refined approaches like MRD assessment are increasingly needed. Therefore, the concept of MRD assessment in hematologic malignancies and its potential application in veterinary oncology is explored as follows.

##### 1) MRD: concept and clinical importance

MRD assessment serves as an important indicator of disease status in hematologic malignancies and represents a key area in which genetic mutations can be clinically applied. MRD refers to the presence of malignant cells remaining below the detection limit in patients with complete remission. These cells retain the genetic phenotype of the original tumor cells. Initiating preemptive treatment in patients with detectable MRD offers clinically meaningful benefits. Treatment at the MRD stage aims to enhance drug sensitivity and effectively suppress relapse by minimizing changes in the tumor microenvironment and clonal complexity. MRD assessment is used to determine the need for further chemotherapy after complete remission, adjust treatment intensity, and predict relapse at an early stage [73,74]. For example, in the treatment of human acute lymphoblastic leukemia (ALL), MRD assessment is incorporated into standard therapy following first-line multi-agent chemotherapy. Alternative treatments like blinatumomab are considered for patients with B-ALL and persistent MRD. In patients with T-ALL, if MRD persists within the first 4 months of treatment or after consolidation therapy, treatment intensity may be increased, or allogeneic hematopoietic stem cell transplantation may be considered [74]. In patients with chronic myeloid leukemia carrying the BCL–ABL fusion kinase, insufficient initial response to TKI therapy or rising MRD levels may suggest the emergence of resistance mutations, prompting the consideration of switching to a second- or third-generation TKI [73]. Although substantial evidence demonstrating that MRD-guided therapy is superior to empirical treatment remains limited, MRD monitoring offers high clinical value by allowing intervention before overt clinical relapse, helping to prevent systemic deterioration and providing an objective measure for assessing patient status. Accordingly, interest in MRD is expected to grow in the field of veterinary oncology as well. The key to MRD assessment lies in the identification of appropriate biomarkers, which must be coupled with the application of precise detection methods. NGS-based analyses and tumor-specific mutation panels have been utilized in biomarker discovery, and such approaches may be extended to MRD assessment in veterinary medicine based on mutations reported in canine cancer.

##### 2) MRD assessment in canine hematologic malignancies

Reports on MRD assessment in dogs focus on B-cell lymphoma, using PCR for antigen receptor rearrangements (PARR) [75,76], ASO-PCR [77,78,79,80], and flow cytometry (FC). In one study of dogs with DLBCL, PARR detected residual cells in most relapsed cases, whereas FC showed lower sensitivity. Combining PARR and FC provided the strongest correlation with relapse and survival, although FC alone had limited value, as MRD-negative dogs also relapsed [76,81].

A more sensitive approach for MRD assessment is a quantitative analysis using clone-specific real-time PCR (qPCR) with customized primers for each patient. Using this method, changes in MRD levels during chemotherapy were closely associated with prognosis [77,78]. Dogs achieving molecular remission showed a median remission of 400 days, compared to only 30 days in those with high MRD levels [77]. MRD negativity at week 11 predicted longer survival and higher completion rates of the UW-25 protocol [78]. These findings suggest that even in cases of clinical remission, MRD status should be considered when determining treatment strategies following initial induction therapy. Moreover, MRD assessment has substantial clinical value in predicting early relapse and guiding preemptive intervention. qPCR-based monitoring revealed that MRD elevation preceded relapse by a median of 42 days in most cases [78]. However, conventional primers risk nonspecific amplification, especially in lymph node aspirates, and MRD signals were sometimes observed even in remission. To address this issue, hairpin-structured primers have been proposed [79,80]. In a study using hairpin-structured allele-specific primers to assess MRD levels in the blood of dogs with B-cell lymphoma, MRD positivity preceded clinical relapse by 16–126 days, whereas MRD negativity was maintained in dogs with long-term remission [79]. However, ASO-PCR requires patient-specific reagents and assay conditions, and its ability to track subclonal evolution during treatment is limited, which may result in false-negative findings [74].

Overall, MRD assessment in veterinary oncology relies on PARR and ASO-PCR for relapse monitoring and prognosis, whereas FC remains a supplementary tool due to limited sensitivity.

##### 3) MRD detection techniques: multiparametric FC (MFC), PCR, and NGS

In human leukemia and multiple myeloma, MRD monitoring is routinely integrated into treatment and is evaluated using a combination of MFC, PCR (qPCR, ddPCR), and NGS (Table 1) [74,82,83]. MFC, which uses 6–9 fluorochromes, is rapid and cost-effective but has the lowest sensitivity and requires skilled technical expertise compared to other methods. Mutation-targeted PCR is highly sensitive and standardized; however, it is only applicable to patients who carry the corresponding target gene. In contrast, NGS allows for the simultaneous analysis of multiple clones with extremely high sensitivity, but it is limited by high cost, long turnaround time, and complex data analysis. qPCR and ddPCR target gene mutations, including fusion transcripts like BCL–ABL, whereas ASO-qPCR evaluates MRD based on specific IG or TR V(D)J sequences. With recent advances in NGS technologies, NGS-based MRD evaluation has garnered attention for its ultrahigh sensitivity down to 10^−6^, its ability to comprehensively detect a broad range of genetic alterations, and its capacity to track clonal expansion throughout disease progression. This technique overcomes the traditional limitation of requiring bone marrow-based testing by enabling concordant detection between peripheral blood and bone marrow samples [72]. Accordingly, the NGS-based ClonoSEQ assay has been approved by the United States FDA for MRD assessment in patients with ALL. In a clinical analysis of patients with B-ALL, 38% of those classified as MRD-negative by MFC were MRD-positive according to NGS, confirming its superior sensitivity [84]. Additionally, patients with minimal NGS-based MRD levels of ~10^−4^, which were previously difficult to classify using conventional PCR, had a worse prognosis than that of MRD-negative patients [85]. A bone marrow MRD level of ≥ 10^−4^ before allogeneic stem cell transplantation is associated with a higher likelihood of transplant failure, but traditional PCR and MFC lack sufficient sensitivity when evaluating peripheral blood. In contrast, NGS-based MRD analysis enabled the quantitative prediction of post-transplant relapse risk, showing that patients with MRD >10^−4^ had a 7.7-fold higher relapse rate, and those with MRD ≥ 10^−6^ in post-transplant peripheral blood had a 100% relapse rate. On average, MRD positivity was detected 89 days before clinical relapse [86].

**Table 1:** Comparative summary of MRD detection platforms relevant to veterinary oncology practice

Technology	Approximate analytical sensitivity (limit of detection)^a^	Main advantages	Main limitations
Multiparametric flow cytometry	10^−3^–10^−4^	Rapid results (2–4 h); real-time analysis; cost-effective	Lower sensitivity; requires fresh samples; requires technical expertise
PCR-based methods (qPCR/ASO-PCR)	10^−4^–10^−5^	Highly sensitive; enables standardized, patient-specific tracking	Requires a known target sequence; false negatives due to clonal evolution; requires custom reagents
Droplet digital PCR (ddPCR)	10^−5^–10^−6^	Highly sensitive; absolute quantification; detection of rare variants	Complex workflow; higher cost; low sample throughput; limited multiplexing; unable to track clonal evolution
Next-generation sequencing (NGS)	Down to 10^−6^	Highest sensitivity; comprehensive multi-clone tracking	Limited availability in veterinary practice; long turnaround time; high cost; lack of validated veterinary-specific pipelines

The table summarizes the approximate analytical sensitivity, principal advantages, and major limitations of currently used MRD detection approaches, including multiparametric flow cytometry, PCR-based methods (qPCR/ASO-PCR), and droplet digital PCR (ddPCR).

ASO-PCR, allele-specific oligonucleotide polymerase chain reaction; ddPCR, droplet digital polymerase chain reaction; MRD, minimal residual disease; NGS, next-generation sequencing; PCR, polymerase chain reaction; qPCR, quantitative polymerase chain reaction.

^a^Sensitivity is expressed as the approximate limit of detection (fraction of malignant cells detectable; e.g., 10^−6^ denotes one tumor cell among 10^6^ normal cells). Lower values indicate higher analytical sensitivity.

As genomic profiling advances, NGS-based MRD assessment is expected to guide treatment intensity, relapse prediction, and transplant outcomes in dogs, paralleling human oncology. Particularly, mutation-targeted MRD strategies using NGS or cell-free DNA (cfDNA), although still lacking veterinary validation, represent a promising direction for future research [74].

### Liquid biopsy

Detecting mutations in body fluids is referred to as liquid biopsy, which holds great promise for broad applications in hematologic malignancies and solid tumors. Liquid biopsy can target various samples, including blood, urine, and cerebrospinal, ascites, and pleural effusion fluids (Fig. 2). Although tissue biopsy is still the standard to confirm mutations, liquid biopsy can serve as a highly effective diagnostic tool, especially in patients for whom invasive procedures are not feasible or in cases requiring repeated assessments, e.g., recurrence monitoring. Liquid biopsy targets a wide range of tumor-associated molecules present in body fluids, including circulating tumor DNA (ctDNA), circulating tumor cells (CTCs), exosomes, tumor-educated platelets, microRNA, long noncoding RNA, and circular RNA [14]. ctDNA, released from tumors or CTCs through apoptosis or necrosis, typically consists of short fragments of < 200 bp and has a very short half-life of < 2 h, offering an advantage for the real-time assessment of systemic tumor burden [87].

**Fig. 2:**
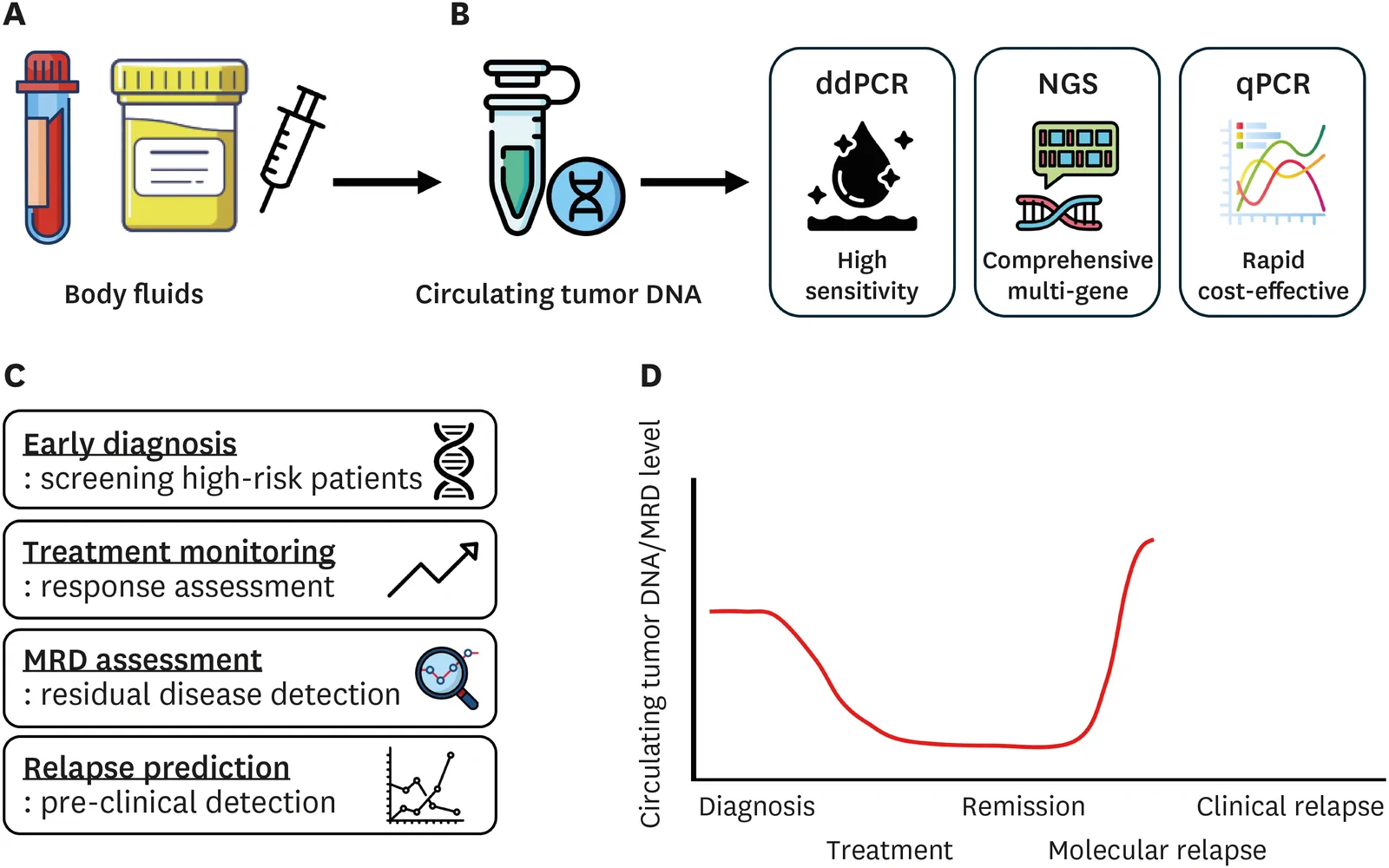
Liquid biopsy workflow and clinical utility of circulating tumor DNA (ctDNA) in canine cancer. (A) Sample collection from body fluids, such as blood, urine, and effusions, through minimally invasive procedures. (B) Cell-free DNA extraction and ctDNA analysis using high-sensitivity platforms: droplet digital PCR (ddPCR), next-generation sequencing (NGS), and quantitative PCR (qPCR). (C) Clinical applications, including early diagnosis, treatment response assessment, minimal residual disease (MRD) detection, and relapse prediction. (D) Schematic dynamics of ctDNA/MRD levels across the clinical course. Following treatment, levels decline during remission and rise at molecular relapse, which precedes clinical relapse and provides a window for early intervention. The curve is schematic and does not depict measured values (y-axis, relative ctDNA/MRD level in arbitrary units; x-axis, time). ctDNA, circulating tumor DNA; ddPCR, droplet digital polymerase chain reaction; MRD, minimal residual disease; NGS, next-generation sequencing; qPCR, quantitative polymerase chain reaction.

Liquid biopsy can serve as a complementary tool to conventional diagnostic methods or as their substitute in specific, limited situations. A representative example is non-small cell lung cancer (NSCLC), a common cancer in humans. In NSCLC, liquid biopsy shows high concordance with tissue testing and better reflects tumor heterogeneity [88]. According to the National Comprehensive Cancer Network (NCCN) guidelines, ctDNA cannot replace histology but may be used when tissue is unobtainable; the 2022 European Society for Medical Oncology (ESMO) guidelines recommend ctDNA analysis when tissue genotyping is delayed, risky, or anatomically challenging [89,90]. Thus, liquid biopsy can be applied throughout the entire clinical course of cancer, from diagnosis to treatment, and holds potential as a surveillance tool for high-risk individuals. Numerous studies have demonstrated its diagnostic accuracy across various cancer types and stages. For example, the CancerSEEK model combined ctDNA and protein markers to detect eight cancers with stage-dependent sensitivity (43% in stage I, 78% in stage III) and > 98% specificity [91]. In the 2020 PanSeer study, machine learning was applied to 477 genomic regions to predict cancer development in five major tumor types, identifying individuals who developed cancer within 4 years with a sensitivity of 95% [92]. In early-stage colorectal, breast, lung, and ovarian cancers, somatic mutations were detected in blood with high concordance to tumor tissue [93]. Similarly, *BRAF* V600E in melanoma showed strong tissue–plasma concordance, and baseline ctDNA levels by ddPCR correlated with survival outcomes [94].

Conventional imaging studies may delay the detection of new lesions associated with tumor progression, whereas liquid biopsy allows for real-time monitoring of mutation levels during surgical resection or chemotherapy by tracking whether the number of detected mutations continuously decreases or remains unchanged, which in turn enables the evaluation of recurrence risk and prognosis [95]. The detection rates of ctDNA by digital PCR were higher in metastatic than in localized tumors and were correlated with stage [96]. In metastatic colorectal cancer, ctDNA levels typically declined postoperatively, but persistent ctDNA predicted a markedly higher 1-year recurrence rate [97].

Liquid biopsy can also be used to select therapeutic strategies and evaluate drug resistance. For example, in melanoma, a low basal mutant *BRAF* ctDNA concentration was associated with a better response rate and longer PFS when treated with the *BRAF* inhibitor dabrafenib [98], and ctDNA levels corresponded with clinical and imaging outcomes in patients with melanoma receiving immune checkpoint inhibitors [99]. In NSCLC, epidermal growth factor receptor (*EGFR*) mutations provide important information for predicting drug resistance and TKI response [100].

#### Liquid biopsy in veterinary oncology: from screening to monitoring

Building on key somatic mutations, this section highlights clinical applications of liquid biopsy in canine oncology. Owing to its noninvasive, low-risk, and repeatable nature, ctDNA analysis is now emerging in veterinary oncology. However, clinical standards, target selection, and scope of use remain undefined due to insufficient data.

Although initial attempts focused on distinguishing malignant tumors based on cfDNA concentration [101] or fragment length [102,103], more recent attention has shifted toward liquid biopsy using NGS or PCR targeting tumor-specific somatic mutations. Although histopathologic diagnosis and tissue-based sequencing remain the diagnostic gold standard, an important advantage of liquid biopsy is its ability to reflect tumor heterogeneity [104]. Several studies showed that more somatic mutations were detected in cfDNA than in the corresponding tumor tissue [105,106]. These findings suggest that liquid biopsy may serve as a complementary diagnostic tool to overcome the limitations of conventional tissue biopsy.

#### ctDNA as a biomarker for monitoring tumor burden and relapse

Currently, the most extensive study applying liquid biopsy in dogs is the CANDiD Study, a multicenter NGS-based liquid biopsy project conducted across 41 institutions in six countries, enrolling over 1,000 dogs. This study aimed at early multicancer detection using blood-based analysis, including somatic mutations in 50 major cancer-related genes like *TP53*, *KRAS*, *BRAF,* and *PIK3CA*. The cohort included not only dogs with various malignant tumors but also those with non-neoplastic comorbidities and healthy controls. The ctDNA-based test showed 54.7% sensitivity, 98.5% specificity, and stage-dependent detection (16.1% in stage I vs. 89.3% in stage IV), with the highest rates in hemangiosarcoma (89.2%) and lymphoma (81.6%) [107]. Using the same platform, a 2023 prospective study of 359 dogs confirmed detection in 32% of localized, 48% of preclinical, and 84% of metastatic tumors, whereas 61.5% of dogs later diagnosed with cancer were ctDNA-positive at health screening.

A balanced assessment of the CANDiD study is essential to avoid overstating current clinical readiness. While the 54.7% overall sensitivity and 98.5% specificity represent a meaningful proof of concept, several critical limitations must be acknowledged. First, the 16.1% detection rate for stage I tumors indicates that liquid biopsy currently cannot serve as a standalone screening tool for early-stage or asymptomatic disease—the clinical setting where it would be most impactful. Clinicians must counsel owners accordingly, as a negative ctDNA result does not exclude malignancy, particularly in early-stage patients. Second, the study identified cases of ctDNA detection in dogs without confirmed tumors, raising unresolved concerns about false positives, the clinical significance of low-level ctDNA signals, and the risk of prompting unnecessary diagnostic procedures or treatment. Third, stage-dependent performance (32% detection in localized disease vs. 84% in metastatic disease) mirrors limitations observed in human multi-cancer early detection trials, suggesting that tumor burden—rather than the specific detection platform—may be the primary limiting factor. These constraints indicate that liquid biopsy in veterinary oncology is most appropriately positioned as a complementary monitoring tool in diagnosed patients, rather than a primary screening modality, until sensitivity in early-stage disease is substantially improved through higher-coverage sequencing or tumor-informed approaches.

Interestingly, among 13 dogs who visited the hospital for routine health screening but were later diagnosed with cancer, 61.5% were ctDNA-positive [108]. These studies support the clinical utility of liquid biopsy as a screening tool for high-risk individuals at the preclinical stage. Postoperative ctDNA persistence doubled the recurrence risk within 6 months, independent of margin status, and molecular relapse preceded clinical relapse by a median of 168 days in several dogs [109]. Similarly, a study on osteosarcoma showed that an increase in the tumor fraction of cfDNA was observed prior to clinical recurrence, suggesting the potential of liquid biopsy in monitoring relapse and treatment response [105].

#### Single tumor applications: hemangiosarcoma, CMTs, and others

As an example of ctDNA analysis focused on a single tumor type, a 2022 study utilized shallow WGS to evaluate copy number variants in plasma ctDNA, distinguishing between malignant and benign splenic lesions. The average presurgical ctDNA tumor fraction was significantly higher in malignant (11.2%) than in benign (3.2%) cases, with a high diagnostic accuracy reflected by an area under the receiver operating characteristic curve of 0.84. Additionally, the ctDNA fraction decreased after surgical resection in both groups [110].

In an earlier 2013 study of CMTs, sequencing and cfDNA-based ddPCR revealed heterogeneous alterations but consistently identified a proximal *PFDN5* deletion on CFA27. Expanded analyses of 20 cases confirmed this deletion in 50%, and ctDNA was detectable in plasma, including one case where cfDNA persisted 1 year after surgery and preceded pulmonary metastasis [111]. In another study evaluating *BRCA2* mutations in dogs with CMTs, single-nucleotide polymorphisms or insertion–deletions (indels) primarily located in exon 11 were identified in tumor tissue and assessed using ctDNA. Dogs diagnosed with tumors and subsequently undergoing surgical removal showed higher ctDNA levels than those in the control group [112]. Comparative oncology has revealed shared mutations in CMT and human breast cancer, including *GATA3, CCNE1, ERBB2*, and *PIK3CA* mutations. *CCNE1* mutations were detectable in the plasma of all canine patients, whereas *PIK3CA* mutations were linked to recurrence and *GATA3* mutations to remission, with *MAP3K1* and *PMS2* mutations proposed as predictors of treatment response [106]. *PIK3CA,* frequently mutated in human breast cancer, was also frequently mutated in CMT (43.1%), particularly in benign tumors, suggesting an early oncogenic role [113,114]. Based on this, ddPCR analysis of cfDNA showed high concordance with NGS (95.2%) and tumor tissue (84.2%), highlighting *PIK3CA* as a strong liquid biopsy target in CMT [115].

#### Alternative liquid biopsy biomarkers: exosomes, CTCs, and nucleosomes

In addition to somatic mutations, various liquid biopsy approaches detect nucleosomes, exosomes, and CTCs in plasma for tumor diagnosis. In splenic tumors, nucleosome levels were significantly higher in malignant cases, especially hemangiosarcoma, with a diagnostic sensitivity of 67.9% and specificity of 71.1% [116]. In a study involving dogs with lymphoma, serum levels of extracellular vesicles were higher in dogs with complete response than in those with progressive disease and correlated with survival. This study also suggested the potential to predict chemotherapy response and identify nonresponders at an early stage through oncomiR pattern analysis [117].

## DISCUSSION

Growing evidence has defined cancer as a genomic disease, driving the adoption of precision oncology in veterinary medicine. Comparative oncology has revealed oncogenic drivers and biomarkers shared between human and canine tumors. In dogs, somatic mutation profiling has enabled diagnostic and prognostic applications, while MRD assessment using ctDNA and liquid biopsy tools, including ddPCR and NGS, is gaining traction for monitoring. Despite this progress, veterinary cancer genomics remains in its early stages and requires further clinical integration. Accordingly, we propose future directions to enhance biomarker discovery and clinical integration in veterinary oncology, building on current insights for translation into somatic mutation-based diagnostics and the use of liquid biopsy across tumor types.

### Evidence-based technology selection framework

In contrast to human oncology where WGS is increasingly feasible, the practical realities of veterinary clinical settings necessitate a tiered approach to technology selection. Based on the evidence reviewed herein, we propose the following framework: for initial diagnostic profiling: TS panels (e.g., OncoK9, SearchLight DNA, FidoCure) currently offer the optimal balance of clinical utility, turnaround time, and cost-effectiveness for most veterinary practitioners. They are most appropriate when the clinical objective is identification of actionable mutations in known cancer-related genes; for longitudinal monitoring of known mutations: ddPCR is preferred over NGS-based approaches due to its superior sensitivity for rare variants in liquid biopsy samples, absolute quantification capability, and lower cost per assay. However, its limited multiplexing capacity and inability to detect novel clonal variants must be considered during long-term monitoring; for comprehensive genomic profiling or research applications: WGS provides the most complete landscape but remains impractical for routine clinical use due to cost, computational requirements, and the lack of standardized veterinary-specific analysis pipelines. No single platform is universally superior; the appropriate choice depends on the clinical objective, available infrastructure, and stage of disease.

### Multigene panel testing for early and complementary cancer detection

Tumor-specific rare biomarkers can increase specificity, but due to their low prevalence and lack of universality, a risk of false positives exists. Moreover, for tumors with relatively low clinical burden, the sensitivity is markedly reduced [91,96]. To address these issues, a comprehensive approach using multigene panel testing has been proposed [118,119]. In the diagnostic field, NGS-based diagnostic panels, including multiple gene mutations, have been developed [120]. For prognostic prediction, Oncotype Dx®, the most widely used test in the United States to assess breast cancer gene expression, is a representative example. This profiling test integrates the expression levels of 21 genes with clinical data to help determine the risk of breast cancer recurrence and the need for adjuvant chemotherapy [121].

In the Canine Oncopanel study, an NGS diagnostic panel targeting 283 cancer genes was constructed, and putative driver mutations were detected in more than 90 percent of the samples [122]. Similarly, the CANDiD Study demonstrated the ability to detect cancer signals with high specificity based on more than 50 gene mutations [107]. A few veterinary diagnostic companies have developed their own gene panels and currently offer genomic profiling services. However, the major oncogene and tumor suppressor gene mutations included in these panels were primarily selected based on human tumor data and reflect the mutational characteristics of tumors prevalent in humans, which presents a limitation. Therefore, further studies are warranted to identify gene biomarkers associated with tumor-specific or shared oncogenic pathways in dogs.

Even in human medicine, limitations exist for confirming a diagnosis based solely on detected gene mutations, and its use is recommended as a complementary or confirmatory method alongside conventional pathological diagnosis [97]. Accordingly, further studies are needed to evaluate whether integrating gene mutations into cancer screening can contribute to improved diagnostic accuracy in veterinary oncology.

### Species-specific biological constraints in translating MRD and ctDNA paradigms

Translating MRD and ctDNA monitoring paradigms from human to canine oncology requires explicit acknowledgment of biological and clinical constraints that are often understated in comparative reviews.

ctDNA shedding rates: The proportion of ctDNA in total cell-free DNA varies substantially not only by tumor type and stage—as demonstrated in the CANDiD study (16.1% detection at stage I vs. 89.3% at stage IV)—but also potentially across species. Lower tumor burden in early-stage canine cancers, combined with less-characterized shedding kinetics in dogs, often results in ctDNA levels below the limit of detection for standard NGS. This necessitates the use of ultra-deep sequencing or patient-specific ddPCR assays for reliable early-stage detection.

Breed-associated genetic architecture: Unlike the relatively uniform human population context in which MRD assays were developed, canine genetics are shaped by closed breeding systems that create breed-specific mutational backgrounds. This genetic stratification means that universal mutation panels derived from human oncology may have significantly different sensitivity and specificity across breeds—a factor that must be prospectively evaluated in breed-stratified veterinary studies.

Serial sampling feasibility: Human MRD protocols typically involve scheduled, protocol-mandated blood draws at defined treatment timepoints. In veterinary practice, owner compliance, patient temperament, financial constraints, and the absence of standardized monitoring protocols represent practical barriers to the serial sampling required for longitudinal ctDNA monitoring.

Canine lifespan and treatment decision timelines: The shorter natural lifespan of dogs compresses the clinical timeline within which MRD-guided therapeutic adjustments can be implemented. MRD positivity detected 89 days before relapse—a meaningful lead time in human leukemia—represents a proportionally larger fraction of a dog’s remaining lifespan, which may paradoxically narrow the window for effective preemptive intervention unless treatment protocols are adapted accordingly.

### Mutation-guided therapeutics and tumor-agnostic therapy

Gene mutations can impact therapeutic strategies. For instance, the use of TKIs is determined by the presence or absence of *EGFR* mutations in human lung cancer, as targeted therapies offer little clinical benefit in patients without such mutations [123]. According to the 2021 guidelines of the International Association for the Study of Lung Cancer (IASLC), initial ctDNA testing is recommended to identify the EGFR T790M mutation in cases of acquired resistance to EGFR TKIs, for which third-generation TKIs are the treatment of choice [100].

Although the examples above illustrate mutation-guided therapies within a specific cancer type, recent advances in precision oncology have driven a paradigm shift toward tumor- or tissue-agnostic approaches. This therapy is no longer guided by the anatomical origin of the tumor but rather by shared molecular alterations across diverse malignancies. Several targeted and immune therapies have received FDA approval under this paradigm, with pembrolizumab (Keytruda®; Merck & Co., Inc., USA) pioneering this approach. As a programmed cell death-1 inhibitor, pembrolizumab is approved for patients with unresectable microsatellite instability-high or mismatch repair-deficient solid tumors, regardless of tumor type [124]. Similarly, ctDNA containing known somatic cancer gene variants is an actively studied biomarker for predicting and evaluating responsiveness to immune checkpoint inhibitors, irrespective of tumor type [125]. In veterinary clinical practice, targeted therapies are often administered empirically to unresolved malignant tumors without reference to tumor-specific molecular features or a defined tumor type, which can result in missed opportunities for effective treatment or unnecessarily increased medical costs. Despite the rapid introduction of targeted therapies and immune checkpoint inhibitors into clinical settings, standard guidelines are still lacking regarding which drug should be applied to which patient at what time. Therefore, research on tumor biomarkers and liquid biopsy holds great potential for clinical application as a basis for drug selection and adjustment, as well as continuous evaluation of therapeutic response.

AI as an enabler of veterinary precision oncology: the value of AI in this context is not as a standalone topic, but as a critical enabler of the genomic approaches described throughout this review. In veterinary oncology, where training datasets are limited compared to human oncology, AI-based approaches face challenges that must be addressed. Machine learning models for canine cancer currently rely on small, often single-institution cohorts, limiting their generalizability across breeds, geographic regions, and clinical settings [126].

However, emerging applications are directly relevant to the genomic framework presented here: AI-assisted variant classification may help distinguish driver from passenger mutations in canine-specific genomic contexts where manual curation is limited by the relative scarcity of functional annotation data [127,128]. Multimodal integration of genomic mutation profiles with clinical outcomes data—analogous to the cBioPortal approach in human oncology—could accelerate the validation of canine-specific biomarkers if applied to multicenter veterinary datasets [122,129].

Realizing these benefits requires the foundational infrastructure of universal data sharing: accumulation of annotated canine tumor genomic profiles, standardized data formats, and open-access repositories analogous to TCGA for veterinary oncology. Without these prerequisites, AI applications in veterinary precision oncology will remain limited in scope and clinical impact [130].
